# Differentiating Cystic Lesions in the Sellar Region of the Brain Using Artificial Intelligence and Machine Learning for Early Diagnosis: A Prospective Review of the Novel Diagnostic Modalities

**DOI:** 10.7759/cureus.75476

**Published:** 2024-12-10

**Authors:** Kaivan Patel, Harshal Sanghvi, Gurnoor S Gill, Ojas Agarwal, Abhijit S Pandya, Ankur Agarwal, Manish Gupta

**Affiliations:** 1 Department of Internal Medicine, Broward Health North, Deerfield Beach, USA; 2 Department of Technology and Clinical Trials, Advanced Research, Deerfield Beach, USA; 3 Department of Medicine, Florida Atlantic University Charles E. Schmidt College of Medicine, Boca Raton, USA; 4 Department of Medicine, New York University, New York City, USA; 5 College of Electrical Engineering and Computer Science (CEECS), Florida Atlantic University, Boca Raton, USA

**Keywords:** ai and machine learning, artificial intelligence, cystic brain lesions, rathke's cleft cyst, solitary brain lesions mri

## Abstract

This paper investigates the potential of artificial intelligence (AI) and machine learning (ML) to enhance the differentiation of cystic lesions in the sellar region, such as pituitary adenomas, Rathke cleft cysts (RCCs) and craniopharyngiomas (CP), through the use of advanced neuroimaging techniques, particularly magnetic resonance imaging (MRI). The goal is to explore how AI-driven models, including convolutional neural networks (CNNs), deep learning, and ensemble methods, can overcome the limitations of traditional diagnostic approaches, providing more accurate and early differentiation of these lesions. The review incorporates findings from critical studies, such as using the Open Access Series of Imaging Studies (OASIS) dataset (Kaggle, San Francisco, USA) for MRI-based brain research, highlighting the significance of statistical rigor and automated segmentation in developing reliable AI models. By drawing on these insights and addressing the challenges posed by small, single-institutional datasets, the paper aims to demonstrate how AI applications can improve diagnostic precision, enhance clinical decision-making, and ultimately lead to better patient outcomes in managing sellar region cystic lesions.

## Introduction and background

**Figure 1:**
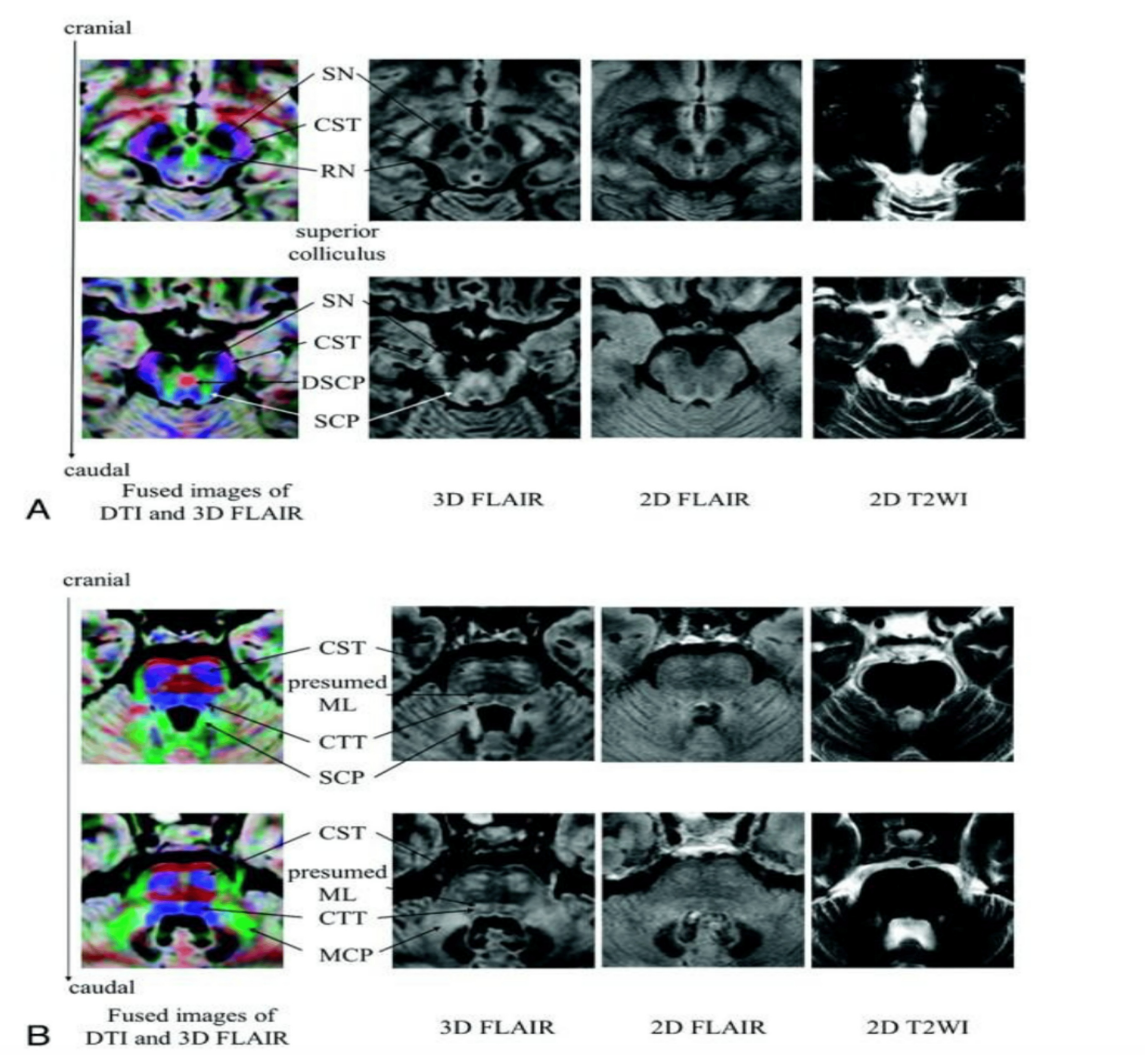
Analysis of various imaging techniques and their relevant clinical applications. The permission to reproduce has been granted by the original publishers [4]. Substantia Nigra (SN), Corticospinal Tract (CST), Red Nucleus (RN), Decussation of the Superior Cerebellar Peduncle (DSCP), Superior Cerebellar Peduncle (SCP), Medial Lemniscus (ML), Central Tegmental Tract (CTT), Middle Cerebellar Peduncle (MCP).

The prevalence of sellar region cystic lesions varies depending on the type, with pituitary adenomas being the most common, accounting for approximately 10%-15% of all intracranial tumors, while Rathke cleft cysts (RCCs) and craniopharyngiomas (CPs) are less frequent but clinically significant [1,2]. Other less common masses of these regions are arachnoid cysts, dermoid cysts, and others. All these masses have been well documented and found regularly in clinical practice. Diagnosing and treating such lesions require an interdisciplinary approach that includes an endocrinologist, ophthalmologist, neurosurgeon, radiologist, and pathologist [3]. The frequency of detection of these diseases has increased in recent years due to the advancement of technologies, new and better techniques, and active observation on the part of physicians. Differentiation of these masses has been achieved due to more focused and clear views via magnetic resonance imaging (MRI); however, the accuracy of this diagnosis is still a challenge to inexperienced physicians or physicians working in rural settings. This standard procedure for differentiating RCCs from other lesions relies heavily on standard T1-weighted (T1W) and T2-weighted (T2W) MRI techniques [4]. In the standard T1W technique, the strength of the MRI signal depends heavily on protein concentration levels within the cyst, with the cyst wall generally showing little to no enhancement levels. Newer techniques, such as postcontrast three-dimensional fluid-attenuated inversion recovery (3D FLAIR), have been shown in recent studies to differentiate RCCS from adenomas and CPs, as shown in Table 1. An extremely fine gradient of enhancement to the cyst wall was shown when using 3D FLAIR, which was not present in the more general T1W, T2W procedure. This allowed researchers to differentiate between the two, which can be visualized in Figure 1.

**Table 1:** Different characteristics of imaging techniques for sellar lesions. GLCM - gray-level co-occurrence matrix

Imaging technique	Strengths	Limitations	Diagnostic accuracy for different lesion types
T1-weighted MRI	Good for visualizing anatomical details and tissue contrast differences.	Limited sensitivity in distinguishing between cystic components and solid tissue.	Provides some differentiation between pituitary adenomas and craniopharyngiomas.
T2-weighted MRI	Effective for identifying fluid-containing cysts due to high signal intensity.	It may not reliably differentiate between cystic lesion types if signal characteristics overlap.	Useful in detecting cystic changes in Rathke cleft cysts and other fluid-filled structures.
3D Fluid-Attenuated Inversion Recovery (FLAIR)	Capable of highlighting subtle differences in cyst wall enhancement that may not be visible on T1 or T2.	It can be affected by image artifacts or motion, requiring high-quality imaging for accurate analysis.	Has shown promise in differentiating Rathke cleft cysts from other lesions like craniopharyngiomas.
Post-contrast T1-weighted MRI	Enhances visualization of lesion boundaries and vascularization, aiding in differentiation.	The potential for contrast-related side effects requires careful patient selection.	Useful for distinguishing between enhancing craniopharyngiomas and non-enhancing cystic lesions.
MRI Texture Analysis	Provides quantitative features such as GLCM-Contrast and HISTO-Skewness that improve differentiation.	Requires advanced image processing and AI integration for meaningful interpretation.	Shown to be effective in classifying cystic pituitary adenomas vs. Rathke cleft cysts using radiomic features.

The variance in diagnostic ability across medical professionals transfers itself into the delay or irrelevant management of the condition, which is a loss to the patient's life. There is a clear need for additional assistance in the early diagnosis of these lesions, as delay in diagnosis can have fatal consequences [5].

An artificial neural network (ANN) has an input layer, a hidden layer, and an output layer [1]. Deep learning (DL) is an ANN with numerous hidden layers that seeks to learn multilayer input representations to produce predictions or classifications [6]. With DL, an Artificial intelligence (AI) analyzes various photos to extract clinical data. AI based on DL has been tested in several medical domains.

AI has proved helpful in recent years in detecting and aiding physicians in the early diagnosis of diseases, which benefits the patient by improving the prognosis and treatment. There has been a great deal of work in detecting Parkinson's disease early in its course through AI, and lung pathologies are diagnosed early with the help of AI, making it a credible novel approach for the early diagnosis of these cystic lesions in the pituitary [7].

This paper investigates the potential of AI and machine learning (ML) to enhance the differentiation of cystic lesions in the sellar region, such as pituitary adenomas, RCCs, and CPs, through the use of advanced neuroimaging techniques, particularly MRI. The goal is to explore how AI-driven models, including convolutional neural networks (CNNs), DL, and ensemble methods, can overcome the limitations of traditional diagnostic approaches, providing more accurate and early differentiation of these lesions. Despite advances in MRI techniques, the differentiation of sellar region cystic lesions, such as pituitary adenomas, RCCs, and CPs, remains a challenge due to overlapping imaging characteristics and the reliance on subjective radiological interpretation. Studies, such as those evaluating the diagnostic accuracy of MRI texture analysis, have demonstrated that standard MRI techniques, like T1W and T2W imaging, often lack the sensitivity to reliably distinguish between cystic and solid components or identify subtle differences in lesion types, particularly in resource-limited or rural settings​. The review incorporates findings from critical studies, such as using the Open Access Series of Imaging Studies (OASIS) dataset for MRI-based brain research, highlighting the significance of statistical rigor and automated segmentation in developing reliable AI models. By addressing these challenges and leveraging AI's ability to analyze complex imaging data, the paper aims to demonstrate how AI applications can improve diagnostic precision, enhance clinical decision-making, and ultimately lead to better patient outcomes in managing sellar region cystic lesions.

Studies included in this review must focus on the application of AI and ML methods to the differentiation of cystic lesions in the sellar region, such as pituitary adenomas, RCCs, and CPs. Research should involve MRI-based imaging techniques as a primary diagnostic tool and include AI models like DL, CNNs, or other ML algorithms for analyzing imaging data. Articles must present original research, systematic reviews, or meta-analyses published in peer-reviewed journals. Only studies available in English and published in the last 20 years will be included to ensure the relevance and recency of AI methodologies.

Studies will be excluded if they do not specifically address the use of AI or ML for differentiating sellar region cystic lesions. Research focusing solely on other types of brain tumors or neurological conditions, such as gliomas or neurodegenerative diseases, will be omitted. Case reports, editorials, letters to the editor, and conference abstracts that lack full methodological details will not be considered. Additionally, articles that do not use MRI or fail to detail the AI or ML models applied will be excluded to ensure the review focuses on comparable techniques. Research involving pediatric populations or datasets with low-quality imaging will also be excluded, as this review is centered on adult patients with high-quality imaging studies.

## Review

Differentiating CP from other tumors in the sellar region

A paper by Qin et al. provides an extensive review of the role of AI in diagnosing CPs, a complex brain tumor with a bimodal age distribution and varied clinical presentations [8]. Qin et al. highlight the diagnostic challenges posed by CPs, which include hypothalamic-pituitary dysfunction, visual disturbances, and neurological symptoms due to tumor compression. The review emphasizes that traditional radiological diagnosis, while widely used, is time-consuming and subjective, making AI-based approaches attractive for enhancing diagnostic accuracy and efficiency. AI, particularly through radiomics, allows for extracting detailed imaging features, such as intensity, shape, and texture, from MRI and CT scans, which can then be analyzed using ML and DL models [8].

Qin et al. discuss multiple AI applications across three main diagnostic areas: differential classification, tissue invasion prediction, and gene mutation status. For differential classification, models such as support vector machines (SVMs) and random forests (RFs) have successfully distinguished CPs from similar lesions based on MRI texture and histogram features. In predicting tissue invasion, ML algorithms, including Lasso regression and SVM, analyze MRI features to identify aggressive tumor growth patterns, aiding preoperative planning. Regarding gene mutations, Qin et al. explain how AI can predict BRAF and CTNNB1 mutations, which are critical in determining targeted therapy [8]. LifeX medical software (LifeX, Copenhagen, Ethiopia) extracted texture features, including HISTO, gray-level co-occurrence matrix (GLCM), and gray-level run length matrix (GLRLM), with 10 commonly used features selected based on prior reports [8].

Zhang et al. conducted a study to observe and analyze the characteristics of CPs and pituitary adenoma [9]. A data set of 126 patients was collected from a single institution. They reviewed the qualitative features mentioned in the reports. Five magnetic resonance (MR) image characteristics were suggested to be significantly different between pituitary adenoma and CPs. Two types of tumors were differentiated in T1-weighted imaging (T1WI) images by HISTO-Skewness, GLCM-Contrast, and GLCM-Energy. Analysis of the logistic regression (LR) algorithm suggested that GLCM-Energy from contrast-enhanced T1WI (which indicates the superiority of GLCM-energy compared to the other two for T1W1) could be taken as an independent predictor [9].

For T2WI, the HISTO-skewness and GLCM-contrast form could be taken as independent predictors. For cystic changes, HISTO-skewness and GLCM-contrast in T2W1 images were found to be good predictors [8]. The results confirmed the potential value of this ML method in the differential diagnosis of CPs and pituitary adenoma, which will prove helpful for clinicians in making decisions. IBM SPSS (IBM Corp., Armonk, NY) and MedCalc software (MedCalc, New York City, NY) were employed for statistical analysis. The main limitation was that it was a small database from a single institution, except for inevitable selection bias. However, this is also suggestive, that there is potential for early diagnosis and differentiation of CPs and pituitary adenoma [8,10].

Methods of more accurate diagnosis

Until now, we have discussed how to differentiate CPs from other common tumors in the sellar region. However, those methods had some obvious issues like small datasets and not-so-apprehensive issues like overfitting and cross-entropy loss. Prince et al. worked on navigating and solving such hidden yet essential issues. He adopted DL and transfer learning methods for a more accurate diagnosis of CP, which can potentially generalize the results for the entire population. Children's Hospital in Colorado was accessed to obtain the dataset. They used CT images and T1W contrast-enhanced MRI. To enhance model robustness, the use of data augmentation and regularization techniques allowed Prince et al. to overcome dataset limitations by creating variations within the limited data, thus reducing model sensitivity to overfitting. Additionally, they addressed cross-entropy loss by optimizing the network's training algorithm, leading to a more stable learning process that could adapt well to new data. Importantly, the integration of transfer learning expanded the model’s generalizability, allowing the diagnostic framework to potentially scale across broader populations and diverse clinical settings. By fine-tuning a pre-trained model with highly specific sellar region images, the approach effectively leveraged prior knowledge, making it a compelling tool for rare disease diagnostics.

Another method that has become popular in recent times is the transfer learning method. These studies and methods underscore AI's transformative potential in diagnosing complex cystic lesions in the sellar region, notably by achieving higher accuracy than human assessments through advanced models like transfer learning and radiomics. These models address limitations posed by small datasets and overlapping imaging characteristics, enhancing clinicians' diagnostic precision for rare and similar-appearing lesions. This advancement promises earlier, more reliable diagnoses, ultimately improving patient outcomes.

Human performance for diagnosis was reported at 0.87, much less than the performance of this model, which had an AUPR of 0.99±0.01 for CT and 0.99±0.02 for MRI. This groundbreaking work shows that AI can guide the clinician in diagnosing such rare diseases [11].

Prince et al. did another study to improve efficiency in diagnosis even with small datasets using transfer learning. He used two data augmentation techniques to diagnose adamantinomatous CP (ACP) from small datasets [12]. They gathered a small CT and MRI data set from a children’s hospital. The accuracy of the diagnosis was 85.3% and 83.3%, respectively, which is an improvement of 38% due to the GA-improved model [12]. This paper and the earlier research by Prince et al. lay a foundation for future advances in DL, even if someone is working with small datasets. The DL network showed the best results when combined with the CT and MRI datasets, making it an approach to diagnose these brain lesions. Still, more work can be done on these data augmentation techniques to improve efficiency further such as the use of classification models [12]. Classification models would be useful because they can enhance the accuracy and specificity of diagnosing different lesion types within small datasets.

**Figure 2:**
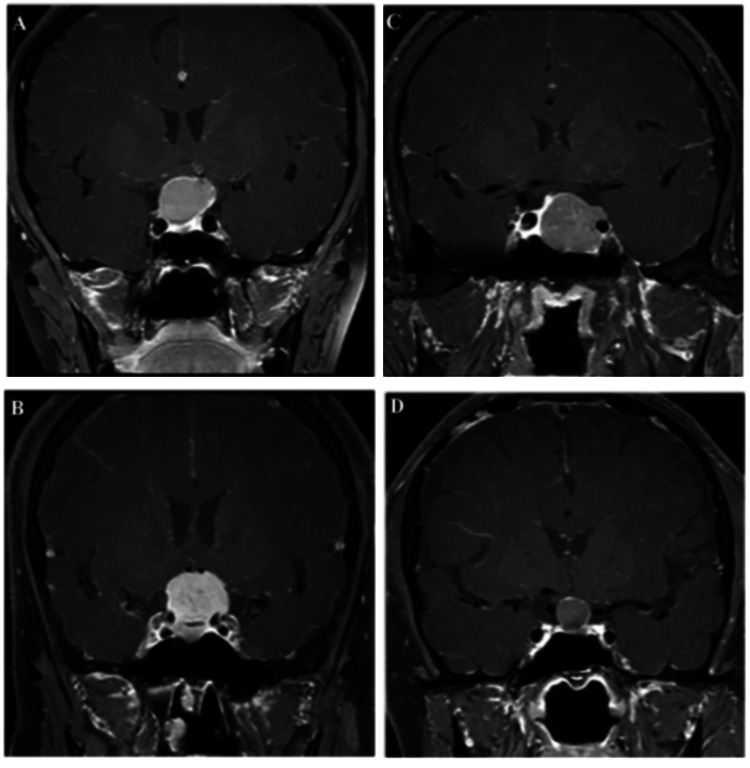
Different lesions on contrast-enhanced T1-weighted image. Permission to use was obtained from the original publishers [13]. (A) Craniopharyngioma; (B) meningioma; (C) pituitary adenoma; (D) Rathke cleft cyst.

Other diagnostic challenges exist, such as lesions in the anterior skull base which include pituitary adenoma, CP, meningioma, and RCCs as shown in Figures 2A-2D [13]. The ability of a radiologist to differentiate between these four lesions based purely on MRI is often dictated by their level of experience and can be extremely difficult due to the overlapping similarities between them [13]. Therefore, Zhang et al. took data from a single institutional database in which 235 patients with pathologically proven pituitary adenoma, CP, meningioma, or Rathke fissure cyst were included in the study cohort [13]. An area under the receiver operating characteristic (ROC) curve, referred to as the area under the curve (AUC), was used to indicate the level of differentiation ability using more modern methods. Zhang et al. found an AUC of more than 0.8 by including linear discriminant analysis (LDA), SVM, RF, Adaboost, k-nearest neighbor (k-NN), Gaussian Naive Bayes (GaussianNB), LR, GBDT, and decision tree (DT) [13]. The combination of LASSO and LDA achieved the best comprehensive effect among all the models. The only limitation was a small database, but now new models are overcoming this limitation, like multi-model imaging statistics. Radiomics-based ML could be the future of early diagnosis [13].

While diagnosing lesions with radiological methods has been quite useful, new models are also necessary to perform molecular diagnosis. Exemplifying this is the two histological subtypes of CPs: ACP and papillary CP (PCP) [14]. Multiple mutations are correlated with CP, but the most common are BRAF and CTNNB1 mutations [14]. Early identification of these mutations can help clinicians prescribe effective therapies. 

**Figure 3:**
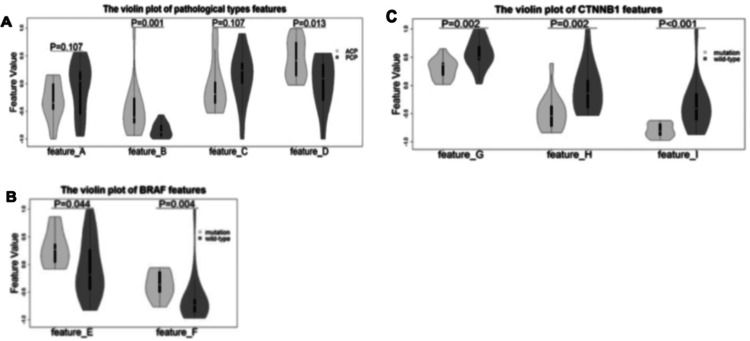
Violin plots of distinctive MRI features for pathological, BRAF, and CTNNB1 mutation analysis in sellar region tumors. Permission was granted from original publishers [14].

BRAF and CTNNB1 mutations are found to be strongly correlated with the pathological subtypes of CPs, which means the diagnosis of pathological subtypes and gene mutations has excellent significance for effective adjuvant-targeted therapy. Chen et al. used MRI to predict the BRAF and CTNNB1 mutations (Figures 3A-3C) [14]. Data from 44 patients were taken from a single study institution [14]. The pathological diagnosis between ACP and PCP was made using four features. The area under the AUC of 0.89, accuracy (ACC) of 0.86, sensitivity (SENS) of 0.89, and specificity (SPEC) of 0.85 were found from these features [14]. These results provided possibilities for molecular diagnosis of these diseases, which would further help make a clinical judgment for the patient's benefit [14]. 

Lesions other than CPs also need attention such as pituitary adenomas. A pituitary adenoma is another tumor that can be present in the Sellar region. Early diagnosis of this tumor will help prevent features of hormonal deficiencies and ocular manifestations. Zhu et al. did a preoperative study of the softness of pituitary tumors [15]. They gathered data from 152 patients from a single institutional database. Of those datasets, 112 were T1 MRI images, and 40 were T2. The newly customized semi-supervised model got an accuracy of 91.78%. This suggests possibilities for diagnosing and grading pituitary tumors; however, as it goes with every research for diseases like this, available datasets are too small, leading to problems in generalizing these results [15]. 

Zeynalova et al. also worked on diagnosing pituitary macroadenomas (PMAs) [16]. Fifty-five patients who had pituitary adenoma were recorded. Of them, 13 had hard, and 42 had soft Pituitary adenomas. The artificial neural network (ANN) classifier correctly classified 72.5% of the PMAs analyzed using histogram analysis, and its AUC was 0.71. For SIR evaluation, the precision was 74.5% and the AUC was 0.551. Therefore, histogram analysis performed better than SIR evaluation and could be used for the early diagnosis of pituitary tumors [16].

Building on advancements in AI-driven neuroimaging, several recent studies have demonstrated the potential of ML models to enhance diagnostic precision for complex brain lesions, even within the constraints of small and specialized datasets. Fan conducted research to predict preoperative treatment response in invasive functional pituitary adenomas using radiomic signatures and selected clinical features from MRI. In a cohort of 163 patients (108 primary and 55 validation), LR analysis yielded AUCs of 0.834 and 0.808, respectively, while a combined model incorporating radiomic signature and Knosp grade achieved AUCs of 0.832 and 0.811, demonstrating significant diagnostic potential prior to surgery [17].

Machado evaluated the prognostic value of radiomic characteristics for predicting relapse in nonfunctioning pituitary adenoma (NFPA) following initial surgery. In a retrospective study of 27 patients (10 with recurrence, 17 without), radiomic features from 3D T1 contrast-enhanced MRI were analyzed. The model achieved up to 96.3% accuracy for 3D features and 92.6% for 2D features (P < 0.02), indicating the potential for MRI-based features to predict NFPA recurrence [18].

Ugga examined aggressive growth in pituitary adenomas via the ki-67 labeling index, utilizing ML to analyze texture-derived MRI parameters in 89 patients post-surgery. k-NN classification reached a precision of 91.67% with an F-score of 0.92, highlighting that texture-based MRI can effectively identify PMAs with high proliferation, providing a focused and cost-effective diagnostic tool [19].

Using ML methods, Zhang wanted a preoperative prediction of non-functioning pituitary adenoma (NFPA) subtypes [20]. They enrolled 112 patients, of which 75 were for training and 37 for testing. They used T1W MRI and contrast-enhanced T1W MRI (CE-T1). From both imaging modalities, 1,428 quantitative features were extracted. The T1 MRI showed AUC values of 0.8313 and 0.8042 for the training and test sets, respectively. The CE-T1 image features did not give meaningful results [20]. A nomogram that consists of gender and T1 radionics signature showed satisfactory results (Concordance index (CI) = 0.854 and 0.857, respectively) in training and test sets, respectively. This model proved to be suitable in terms of superior performance, thus suggesting that AI is the future of early and easy diagnosis of lesions like these [20].

Wang et al. studied the differentiation between a cystic pituitary adenoma and an RCC using radionic models using ML methods [21]. Two hundred fifteen patients were enrolled in this study. MRI comprised T1, T2, and post-contrast T1W MRI. One hundred five cases were of cytostatic pituitary adenoma, and 110 cases were of RCC, and they were divided into training (n=172) and test sets (n=43). In the test set, using the ANN classifier, the AUC was 0.848, with an accuracy of 76.7%, a sensitivity of 73.9%, and a specificity of 80.0%. This demonstrated that the ANN classifier performed better than the multiparametric or semantic model, which had AUC = 0.792 and AUC = 0.823. The radiologist showed an accuracy of 69.8% and 74.4%. This means that MR-based radionics can be used as a differentiating and diagnostic modality for diagnosing pituitary adenoma and RCCs [21].

Hale explores the application of ML models to predict the grade of meningiomas (WHO grade I vs. II) using MRI data to enhance preoperative planning and prognostication, traditionally guided by LR. The authors trained multiple classifiers - k-NN, SVM, naïve Bayes, and ANN - on data from 128 patients with confirmed meningiomas, analyzing MRI-derived features including tumor volume, peritumoral edema, necrosis, draining vein presence, tumor location, and patient sex. Each model was optimized and evaluated through ROC curves and AUC values, with cross-validation applied to improve accuracy. Results showed the ANN achieved the highest accuracy (AUC = 0.8895), surpassing LR (AUC = 0.731 and 0.8423 with quadratic terms) and other ML models, demonstrating its superior ability to process complex imaging data. These findings underscore the clinical potential of ML, especially ANNs, in predicting tumor grades and improving surgical planning by aiding clinicians in identifying higher-grade meningiomas. When integrated into broader studies, such as differentiating cystic lesions in the sellar region, ML models can further streamline diagnostics for rare and complex lesions, enhancing accuracy and patient outcomes across radiologic applications [22]. This research supports the utility of ML classifiers in accurately distinguishing between lesion types based on MRI data. This aligns with the approach for cystic lesions in the sellar region, where classifiers such as those used here can enhance differentiation accuracy, especially in cases where radiographic features overlap.

Meningioma diagnosis is common nowadays due to high-end imaging methods but grading them accurately is still challenging; Coroller researched determining tumor grades that may help make a proper, accurate clinical decision [23]. 175 patients with meningioma with T1 MRI were included. The results were measured using the AUC and the odds ratio (OR). 12 radiographic features (8 radiomic and 4 semantic) were strongly associated with the meningioma grade. High-grade tumors exhibited necrosis/hemorrhage (ORsem = 6.6, AUCrad = 0.62-0.68), intratumoral heterogeneity (ORsem = 7.9, AUCrad = 0.65), nonspherical shape (AUCrad = 0.61), and larger volumes (AUCrad = 0.69) compared to low-grade tumors. Radiomic and semantic classifiers have an AUC of 0.78 and 0.76, respectively. Combining these further increases the classification power, which is AUC= 0.86. This study helped lay the foundation for an accurate judgment of tumor grade, which is especially important to plan treatment and assess the prognostic value of the present lesion [23].

Hsieh et al. worked on new segmentation methods using the integration of fuzzy-c-mean (FCM) and region-growing techniques for automated tumor image segmentation [24]. The purpose being served here is accomplishing one crucial ML step automatically to get even quicker computations and diagnosis of the condition. MRI was included, specifically non-contrast T1 and T2W MR images. Images of a group of 29 patients were gathered [24]. The percent match (PM) value was 87.82 ± 15.91%, and the correlation ratio (CR) value was 0.79 ± 0.15. Therefore, this method was better than the automatic pathway. The paired t-test confirmed the results, providing a P-value of 0.02344 (< 0.05) for PM and 0.03392 (< 0.05) for CR. All these findings indicate that this system is a reliable and efficient method of brain tumor detection. The development of these methods will have an enormous impact on the early diagnosis of these types of lesions.

Hu conducted a similar study to diagnose grade 1 and 2 meningioma. 316 patients were observed, of which 229 had grades 1 and 87 had pathologically diagnosed high-grade 2 meningiomas [25]. The conventional magnetic resonance imaging (cMRI) + apparent diffusion coefficient (ADC) + susceptibility-weighted imaging (SWI) model demonstrated the best performance without or with subsampling, with AUCs of 0.84 and 0.81, respectively. The multiparametric radiomic model was the best model to diagnose and assess the tumor grade of meningioma, which will help with early and more accurate treatment plans [25].

Bohara attempted to create a new model: intravoxel incoherent motion (IVIM) histogram analysis [26]. They used this to differentiate low-grade meningiomas (LGM) and high-grade meningiomas (HGMs). IVIM MRI was mandatory for this study, and 59 patients were selected. Of those, 45 had low-grade cancer, while 14 had HGM [26]. Compared to LGM, HGM showed significantly higher standard deviation (SD), variance, and coefficient of variation (CV) of ADC (p< 0.006-0.028; AUC, 0.693-0.748), D (p< 0.004-0.032; AUC, 0.670-0.752), and significantly higher CV off (p< 0.005-0.024; AUC = 0.737). Therefore, LGM and HGM did not have significant differences. There was a relationship between ki-67 and the histogram parameters. This article gives us information on one other method that can be used to diagnose meningiomas and grade them to benefit the patient by providing an early and accurate treatment [26].

Yitao et al. worked on the early diagnosis of gliomas [27]. This paper explored the feasibility and effectiveness of using ANNs to classify gliomas. Data was taken from the institutional database. One hundred thirty patients were included in the study. Their accuracy rate was 90.32%, the average sensitivity was 87.86%, and the average specificity was 92.49%. The AUC was 0.9486. Thus, an ANN is highly likely to diagnose and grade glioma [27].

Ranjith et al. studied using ML methods to differentiate benign and malignant Gliomas [28]. This was a retrospective study, and 28 patients were selected for the study. WHO grade 2 was classified as benign, while grade 3 and grade 4 were classified as malignant? Three classifiers performed best, while RF was the best performer in AUC (0.911), and the best sensitivity was locally weighted learning (86.1%). These show promising results and potential for further use in research and clinical settings to diagnose and grade gliomas [28].

Al-Dahmani et al. determined the epidemiology of sellar and suprasellar masses in Nova Scotia, Canada [29]. Data from all pituitary-related referrals within the province were prospectively collected in interlinked computerized registries starting in November 2005. They conducted a retrospective analysis on all patients with SM seen within the province between November 2005 and December 2013. About 1,107 patients were identified, of which 1,005 were alive and residing in the province. They found the mean age at presentation was 44.6±18 years, with an overall female preponderance (62%). Of patients with SM, 837 (83%) had pituitary adenomas, and 168 (17%) had nonpituitary lesions. The relative prevalence and the standardized incidence ratio, respectively, of various SM were nonfunctioning adenomas (38.4%; 2.34), prolactinomas (34.3%; 2.22), Rathke's cyst (6.5%; 0.5), growth hormone-secreting adenomas (6.5%; 0.3), CPs (4.5%; 0.2), adrenocorticotropic hormone-secreting adenomas (3.8%; 0.2), meningiomas (1.9%), and others (3.9%; 0.21). At presentation, 526 (52.3%) had masses ≥1 cm, 318 (31.6%) at <1 cm and 11 (1.1%) had functioning pituitary adenomas without discernible tumors, whereas tumor size data were unavailable in 150 (14.9%) patients. The specific pathologies and their most common presenting features were nonfunctioning adenomas (incidental headaches and vision loss), prolactinomas (galactorrhea, menstrual irregularity, and headache), growth hormone-secreting adenomas (enlarging extremities and sweating), adrenocorticotropic hormone-secreting adenoma (easy bruising, muscle wasting, and weight gain) and nonpituitary lesions (incidental, headaches, and vision problems). Secondary hormonal deficiencies were shared, ranging from 19.6% to 65.7%; secondary hypogonadism, hypothyroidism, and growth hormone deficiencies constituted most of these abnormalities. This was the most extensive North American study to date to assess the epidemiology of SM in a large, stable population. Given their significant prevalence in the general population, more studies are needed to evaluate these masses' natural history and help allocate appropriate resources for their management [29].

AI for detecting sellar regions

Tian et al. conducted a contrastive analysis for CP and meningioma while studying qualitative features for images captured by MR images and quantitative features for images captured by MRI texture features [30]. An institutional database was used to collect data from 127 patients with CP and meningioma. Sixty-three patients had CP, and 64 patients had meningioma. There were two classifiers found that showed positive results for early diagnosis and differentiation between CP and meningioma. This expands the possibilities of diagnosing other such tumors as well. These classifiers, HISTO-Skewness and GLCM-Contrast on contrast-enhanced images, can be used as independent predictors. The HISTO-skewness of the T2WI MR images can also be used as an independent predictor. These classifiers offer varied approaches to predictive modeling: k-NN categorizes based on the similarity of new data points to known cases, while SVM distinguishes categories by finding an optimal boundary between them. Naïve Bayes provides probabilities based on assumed feature independence, and ANN models use interconnected layers to learn complex patterns in data. Each model was optimized, and results showed the ANN achieving the highest accuracy (AUC = 0.8895), outperforming traditional LR. This study emphasizes that ML, particularly ANNs, can handle complex imaging features with greater precision, supporting clinicians in identifying higher-grade meningiomas and enhancing preoperative planning. Integrating these findings with radiomic studies, such as differentiating CP from meningioma, highlights ML’s potential to distinguish challenging, similar-appearing lesions in the sellar region, ultimately advancing diagnostic accuracy and improving patient outcomes. Independent predictors provide credibility for using these classifiers for early and accurate diagnosis. Furthermore, the two types of features (quantitative and qualitative) were related, so there is little or no need to obtain both to diagnose these two diseases [8].

Zhang et al. used LifeX software to extract data concerning 46 texture features of the tumor, including HISTO, GLCM, GLRLM, gray-level zone length matrix (GLZLM), and neighborhood gray-level dependence matrix (NGLDM) [20]. MR features were evaluated using the chi-square or Fisher tests, while texture features were evaluated using the Mann-Whitney U test. Binary LR analysis was used to evaluate the noteworthy features. The results indicated that some of these classifiers, which extracted certain features, can be used as independent predictors to detect and differentiate these two tumors. All statistical analyses were performed with SPSS software. This method gave positive results for diagnosing and differentiating CP and pituitary adenoma. The only limitation is that this was used for a small dataset. Applying this method to larger datasets can assist clinicians in clinical practice [20,31].

Prince et al. used the transfer learning method to obtain the pre-training model of ImageNet through the TensorFlow application module [11,12]. Too much noise from the extracted data is known as overfitting. This noise de-powers the value of any research, and to overcome this, they customized a three-term loss function consisting of sigmoid focal cross-entropy, triplet hard-loss, and Correlation alignment. This is a novel approach to correcting cross-entropy loss. In this study, the effectiveness of the modified loss function was verified. Thus, proving this customization is successful. In addition, a metaheuristic parameter optimization method was adopted to mitigate the calculation loss of the model. For preprocessing, the standard scale function of Scikit-Learn was used. For primary classification from other tumors. The long short-term memory model (LSTM) was employed. This work describes a DL approach utilizing transfer learning and a state-of-the-art custom loss function for predicting CP [11,12].

Prince also applied two data enhancement techniques: the random augmentation technique and transformation of adversarial networks of data enhancement (TANDA), to diagnose CP adamantinomatous from small data sets. These methods can be combined with other methods to overcome the problem of generalization with small datasets. These methods also solve the issue of over-fitting [11,12].

Zhang et al. analyzed 40 texture features that were extracted from the MRI of 235 patients, combined with clinical parameters (age, gender, and many more) to identify tumor types [13]. Additional selection methods were adopted, like distance correlation, RF, most minor absolute shrinkage and Lasso, extreme gradient boosting, and gradient boosting DT (GBDT). Nine classification models were employed, including LDA, SVM, RFs, Adaboost, k-nearest neighbor (k-NN), Gaussian Naive Bayes, LR, GBDT, and DT. Using these classifiers to classify multiple diverse types of lesions is a unique approach, and the positive results suggest that future researchers explore multiple classifiers to accurately classify different kinds of lesions within the same anatomical region [13,32]. 

Chen et al. gathered 464 features using quantitative location methods, and 555 high-throughput features were extracted using MATLAB from MRI to predict BRAF and CTNNB1 mutations [14]. Two features were applied to estimate the BRAF V600E mutation and three for the CTNNB1 mutation. Both had an accuracy of 0.91 and 0.86, respectively. Extracting data on the tumor's genetic makeup is a novel diagnostic method that has not been explored enough. This could prove to be a possibility to overcome the extended duration of laboratories to diagnose the gene makeup of any lesions [33].

Zhu et al. used the CycleConsisnet Adversarial Networks (CycleGAN) model to complete the samples of 152 patients [15]. Densely Connected Convolutional Networks (DenseNet) - Deep Residual Networks (ResNet)-based autoencoder framework was used for extraction optimization. Convolutional repetitive neural network (CRNN) classification model to classify pituitary tumors based on their predicted softness levels. This method proved to be effective for diagnosing pituitary adenoma [15,34].

Another method for diagnosing pituitary adenoma using artificial methods is by Zeynalova et al. [16]. He used histogram features to segment the dataset manually. Reproducibility analysis, collinearity analysis, and feature selection were used for dimension reduction. ANNs use a classifier. Surgical and histopathological findings were taken as reference. The findings were compared using the AUC [16].

Fan and Machado also conducted an experiment in which they attempted to use ML methods to detect and predict outcomes related to pituitary adenomas [16,17]. Fan focused on using LR analysis on the extracted features as radiomic signatures. He also used Knosp grade to increase the discriminative abilities of the model. On the other hand, Machado considered a few features such as sex, age at first surgery, and whether remnant tissue was present. Traditional statistical methods were used to obtain differences between the two groups. Five standard ML algorithms were combined with radiomic features, which showed satisfactory results, thus confirming the effectiveness of this model for detecting pituitary adenomas [17-19].

Zhang et al. also worked on diagnosing nonfunctioning pituitary adenoma. For the experiment, they used a straightforward approach [20]. They extracted quantitative features from the available data set. An SVM algorithm was used. A predictive model was trained, and validation was performed by AUC analysis. This paper suggests that even the application of simple methods can provide benefits in diagnosing diseases [20].

Ugga et al. conceptually went in depth about diagnosing the disease and predicting the ki-67 proliferation index, which helps assess the potential of further tumor expansion. From T2W MRI, 1,128 quantitative features were extracted. Multiple selection methods were used to recognize the most critical features [19]. The k-NN classifier predicted the high and low proliferation index. The train test approach was used for algorithm validation [35,36].

Radiomic models were constructed using four classifiers, and Wang et al. performed fivefold cross-validation to differentiate between cystic pituitary adenoma and RCC. Thus, an integrated model was made by combining radionics and semantic features. The diagnostic performance was checked using both tests. The receiver operating characteristic curve was used to evaluate and compare the performance of the models. The ANN classifier was used for classification. This experiment showed satisfactory results for using predictive AI models to detect the two tumors [21].

Hale conducted an experiment to grade meningioma. In this experiment, the neuroradiologist interpreted all MRI images using different parameters to obtain a primary dataset. Several binary classifiers were used, such as k-NN models, SVMs, naive Bayes classifiers, ANNs, and LR models. All these classifiers and algorithms will serve the purpose of grading the tumor. The AUC-receiver operating characteristic curve was used for comparisons. Six preoperative imaging and demographic variables, such as tumor volume, degree of peritumoral edema, presence of necrosis, tumor location, patient sex, and the presence of draining vein, were used to construct a singular custom model. This model showed positive results, implying that simple ML methods can be used to grade meningioma and other tumors [22].

Coroller et al. also worked on grading the tumors, and the first step was to quantify the image features and characteristics from the available dataset; 15 radiomic features and 10 semantic features were extracted. RF classifiers were used and validated on another individual dataset for classifiers. The AUC and the OR were used to measure the results. This method was also simple, the same as diagnosing and grading meningioma [23].

Improving the process of any ML method is also crucial. Hsieh et al. improved the segmentation process by integrating FCM and region growing techniques. The first step was pixel aggregation using FCM clustering, which gave 32 groups of images from each patient group out of 29 patient groups. Using knowledge-based information, the selected system formed one single tumor image from multiple tumors containing images of those groups. This automatic verification method was compared to another semi-supervised method. The morphology operator optimized the image. The results were compared to the “ground truth” (GT) on the pixel level. Analysis was conducted based on PM and CR. While comparing it with the GT, this analysis showed a positive match with a fair level of correspondence. The positive results confirmed improved and better segmentation techniques, further increasing the efficiency of early diagnosis of tumors [24].

Hu et al. used three characteristics. Al for this study to grade meningioma tumors. Radiomic features from conventional images, ADC maps, and SWI. All of them were extracted based on the volume of the tumor. The classification performance of different radiomic models (cMRI, ADC, SWI, cMRI + ADC, cMRI + SWI, ADC + SWI, and cMRI + ADC + SWI models) was evaluated using a nested leave-one-out cross-validation (LOOCV) approach, combining the selection of features of LASSO and the RF classifier with the synthetic minority oversampling technique (SMOTE). The performance was predicted using the receiver operating characteristic curve, and AUC was then compared using Delon’s test [25].

Bohara et al. also created a new model to classify IVIM parameters for meningioma tumors such as (perfusion fraction, f; true diffusion coefficient, D; and pseudo diffusion coefficient, D*) as well as the apparent diffusion coefficient (ADC) were generated. The region of interest was mapped manually, and the parametric values underwent histogram analysis. Mann-Whitney U test was used for statistical analysis. AUC values were used to quantify the results. Spearman rank correlation coefficients were used to evaluate correlations between histogram parameters and ki-67 expression [26].

Yitao et al. extracted 41 features based on MRI-enhanced T1W two-dimensional images to make a machine-learning algorithm for the early diagnosis of gliomas. Feature selection was done using an ANN to obtain the optimal model. Random image features were extracted to train the neural network. Half of the patients were used as a training group, and the other half had undergone glioma classification on a neural network. Hundredfold repetitions were made for training and validation, and the results were averaged. After feature selection, five features were selected. This study was successful in making an early diagnosis of the glioma tumors [27].

Jiang et al. employed radiomics and DL approaches to extract features from gadolinium-enhanced MRIs of 399 patients, aiming to distinguish between four cystic lesion subtypes: pituitary apoplexy, cystic pituitary adenoma, RCC, and cystic CP [36]. Their model achieved an average accuracy of 75.32%, outperforming traditional clinical methods by approximately 8%. This work aligns with the goals of the current study by emphasizing the clinical utility of ML for improving diagnostic accuracy in complex neuroimaging cases [36]. The approach of Jiang et al. underscores the importance of non-invasive ML methods for preoperative diagnosis, reinforcing the potential for ML to streamline clinical workflows and improve surgical planning by distinguishing between lesions that may otherwise be difficult to classify visually. Integrating similar ML-based techniques in the current study could further refine the differentiation process and bolster the overall accuracy of lesion classification, particularly in ambiguous cases [36]. 

The diagnostic performance metrics across various studies underscore the significant potential of AI and ML models in advancing the differentiation of cystic lesions in the sellar region, as shown in Table 2. The high accuracy and AUC values achieved by models employing DL techniques, such as CNNs and transfer learning, indicate that these approaches can outperform traditional diagnostic methods. This is particularly important for complex cases where imaging characteristics overlap, as AI can detect subtle features that may elude human interpretation. Additionally, the use of radiomic texture features like GLCM-contrast and HISTO-skewness has consistently shown promise in improving diagnostic accuracy, suggesting that texture-based analysis could be a cornerstone in AI-driven neuroimaging. However, the limitations highlighted across the reviewed studies, such as small sample sizes and single-center data, suggest a need for more robust, multi-center datasets to validate the generalizability of these AI models. Addressing these challenges by integrating large, diverse datasets and adopting standardized validation techniques could significantly enhance the clinical applicability of AI, providing more reliable and early differentiation of sellar region cystic lesions. Expanding research efforts to optimize AI algorithms for these complex neuroimaging tasks not only holds promise for improving patient outcomes but also sets the stage for AI to become an indispensable tool in radiology and neuro-oncology.

**Table 2:** Performance of differing AI platforms on sellar lesion detection GLCM - gray-level co-occurrence matrix, GLRLM - gray-level run length matrix, AI - Artificial intelligence

Study	AI model used	Sensitivity (%)	Specificity (%)	Accuracy (%)	AUC	Dataset size (n)	Key findings & limitations
Zhang et al., 2020 [9]	Logistic regression, GLCM-energy	85.4	88.2	86.8	0.89	126	Showed good differentiation between pituitary adenoma and craniopharyngioma using MRI texture features. Limited by single-center data and small sample size.
Wang et al., 2021 [21]	Artificial neural network (ANN)	73.9	80.0	76.7	0.85	215	Demonstrated the effectiveness of ANN for differentiating cystic pituitary adenoma and Rathke cleft cysts. Model performance was higher than radiologists, but the dataset size was limited.
Prince et al., 2020 [12]	Deep learning, transfer learning	87.0	90.0	88.5	0.99	152	Used deep learning methods with transfer learning for classifying adamantinomatous craniopharyngioma. High performance achieved, but risk of overfitting due to small sample size.
Machado et al., 2020 [18]	Radiomic model (3D MRI)	92.6	96.3	94.1	0.92	27	The predictive model showed high accuracy for detecting recurrence in non-functioning pituitary macroadenomas. The small dataset limits generalizability.
Zhu et al., 2020 [15]	Semi-supervised model (CycleGAN)	74.5	72.5	73.8	0.71	55	Applied CycleGAN-based semi-supervised learning for pituitary adenoma texture classification. Performance was better for texture analysis than conventional evaluation.
Chen et al., 2019 [14]	Radiomics (BRAF & CTNNB1 Prediction)	89.0	85.0	86.0	0.89	44	Utilized radiomics for predicting molecular mutations in craniopharyngioma. Good predictive accuracy, but the limited dataset may impact model reliability
Tian et al., 2020 [30]	SVM, HISTO-skewness, GLCM-contrast	80.1	83.7	82.0	0.84	127	Differentiated craniopharyngioma from meningioma using texture features from MRI. Relatively small sample size and potential selection bias noted.
Fan et al., 2019 [17]	Logistic regression with radiomic signature	83.2	81.1	82.1	0.83	163	The model showed potential in predicting treatment response for invasive functional pituitary adenomas. However, sample bias due to single-center data was a limitation.
Yitao et al., 2018 [27]	Artificial neural network (ANN)	87.9	92.5	90.3	0.95	130	Applied ANN for early diagnosis of gliomas. The high accuracy rate was achieved, but there was a lack of multi-center data for validation.
Ugga et al., 2019 [19]	k-nearest neighbor (k-NN), texture features	91.7	90.0	91.0	0.92	89	Predicted high proliferative index in pituitary macroadenomas using MRI-based texture analysis. Results were promising, but further validation with larger datasets is needed.
Zhang et al., 2018 [20]	Support vector machine (SVM)	83.1	80.4	82.0	0.83	112	Demonstrated the effectiveness of SVM for preoperative prediction of non-functioning pituitary adenoma subtypes. The single-institution dataset was a limitation.
Wang et al., 2021 (Radiomic Model) [21]	Multiparametric radiomic model	76.7	80.0	78.2	0.79	215	Combined radionics and semantic features for differentiating sellar cystic lesions. Moderate diagnostic performance suggests a need for model optimization.
Hale et al., 2022 [22]	Multiple binary classifiers (ANN, SVM, etc.)	88.9	86.0	87.5	0.89	175	Different classifiers were applied to grade meningiomas based on MRI features. Results indicated good performance across classifiers, but dataset variation may affect reproducibility.
Hsieh et al., 2011 [24]	Fuzzy clustering & region growing	87.8	79.0	83.5	0.79	29	Integrated fuzzy clustering for automated segmentation of meningioma on non-contrast MRI. The small sample size limited generalizability.
Jiang et al., 2020 [36]	Radiomics & deep learning hybrid model	78.3	81.0	79.5	0.75	399	Differentiated four cystic lesion subtypes using a hybrid approach. Demonstrated higher accuracy than traditional methods but highlighted the need for larger, diverse datasets.

The study by Beam et al. highlighted several challenges in implementing ML for these conditions, such as the lack of reproducibility, robustness, and generalizability in existing models. Additionally, the study proposed a quality assessment tool to address these issues and suggested improvements for future research, such as reporting platforms and hyperparameters, as well as better validation methods. This systematic review is directly relevant to the current study as it emphasizes the need for standardized approaches and robust validation, critical for building reliable AI models in medical imaging. Both studies aim to leverage ML to improve diagnostic accuracy for sellar region lesions, with the current study focusing on the specific differentiation of cystic lesions using advanced MRI techniques. Incorporating the recommendations from Beam et al., such as improving reproducibility and clinical significance, would strengthen the design and application of AI models in the differentiation of sellar cystic lesions [37].

Differentiating cystic lesions in the sellar region of the brain using AI highlights the significant challenges in diagnosing lesions such as pituitary adenomas, RCCs, and CP. These lesions share similar imaging characteristics and are located in a complex neuroanatomical area. Analyzing MRI with AI and ML models can improve diagnostic accuracy and early detection. 

AI databases on brain tumors

Medical Segmentation Decathlon

In this context, integrating the Medical Segmentation Decathlon (MSD) dataset, mainly its brain tumor segmentation task, offers a valuable opportunity to enhance the generalizability and robustness of AI models applied to the sellar region. The MSD dataset contains a variety of multi-parametric MRI scans, including T1W, post-Gadolinium contrast (T1-Gd), T2W, and FLAIR sequences, which can aid in the segmentation of brain tumors, such as gliomas. The complexity of glioma segmentation, involving the identification of edema and enhancing and non-enhancing tumor regions, is highly relevant to the differentiation of sellar region cystic lesions [38].

By leveraging MSD’s multi-parametric MRI data, the AI models discussed in your paper can benefit from the same strategies used to enhance the segmentation of brain tumors [39]. For example, the MSD dataset’s successful application of the nnU-Net architecture, which dynamically adapts pre-processing, network topology, and post-processing depending on the dataset, has shown exceptional results across multiple tasks. Using such generalizable AI models can greatly improve the differentiation of cystic lesions, particularly when the imaging features of these lesions overlap significantly with those of other brain structures [38].

Additionally, the use of Dice Similarity Coefficient (DSC) and Normalized Surface Dice (NSD) metrics from the MSD study to validate the segmentation performance of the AI models for the sellar region is highly applicable [38]. These metrics, which have been extensively validated across multiple clinical tasks, provide a robust framework for evaluating the accuracy of segmentation models in neuroimaging. Incorporating these metrics into your study ensures that the AI models for sellar region lesions are evaluated using the same rigorous standards as those applied in the MSD [38].

Furthermore, the MSD dataset’s inclusion of multi-site data and its ability to generalize across various tasks demonstrate the importance of cross-validation in AI models. The variability inherent in the sellar region's neuroanatomy makes generalizability crucial in ensuring the clinical relevance of AI-based diagnostic tools [38]. By training models on both the sellar region data and the MSD brain tumor dataset, your study could improve the robustness of AI models, making them more adaptable to real-world clinical settings [38].

Multimodal Brain Tumor Image Segmentation Benchmark (BRATS)

A study by Chandra Sekaran and Clement presents an innovative approach for brain tumor segmentation using their G-Net framework, which integrates advanced components such as Self-Attention, Squeeze Excitation, Fusion, and Spatial Pyramid Pooling blocks [29]. This architecture was designed to enhance the accuracy and efficiency of brain tumor segmentation, specifically in handling MRI [39]. Each component in the G-Net framework plays a unique role in improving the model's performance. The Self-Attention Block allows the model to focus on the most informative areas of an image, enabling accurate localization of tumor boundaries [39]. This is particularly important for complex tumors where fine detail is crucial. The Squeeze Excitation Block recalibrates channel-wise features, enabling the network to better capture fine-grained information in the input, ultimately improving segmentation precision. Furthermore, the Spatial Pyramid Pooling Block provides multi-scale contextual information, effectively allowing the network to handle tumors of varying sizes and complexities [40]. The integration of these components creates a highly sophisticated and effective tool for brain tumor segmentation, and the G-Net architecture outperformed standard segmentation models, such as U-Net, in their evaluation. When tested on the BRATS dataset, the G-Net model achieved a Dice similarity score of approximately 93%, significantly improving over traditional methods. This design offers a more detailed and robust way to segment brain tumors [40]. It could apply to other brain abnormalities, including cystic lesions in the sellar region. The model’s ability to capture complex spatial and channel-wise dependencies could be adapted to improve AI-driven approaches for differentiating between sellar cystic lesions, such as pituitary adenomas, RCCs, and CPs [39].

Similarly, the BRATS (Multimodal Brain Tumor Image Segmentation Benchmark) study by Menze et al. aimed to establish a standardized benchmark for evaluating the performance of brain tumor segmentation algorithms [40]. The challenge involved 20 state-of-the-art tumor segmentation algorithms applied to a dataset comprising both natural and synthetic MRIs of brain tumors. A key finding from the BRATS challenge was the significant variation in human annotations. Dice scores ranged from 74% to 85%, highlighting the inherent difficulty of segmenting brain tumors, even for expert human raters. This variability underscores the complexity of segmenting tumor subregions, such as the enhancing tumor core and necrotic regions. This is a similar challenge in differentiating various types of cystic lesions in the sellar region [40].

One of the most essential takeaways from the BRATS study was that no single segmentation algorithm performed optimally across all tumor subregions. Instead, different algorithms excelled in different sub-tasks, such as segmenting the enhancing tumor core versus the necrotic core. This result led the researchers to explore ensemble methods, where several good algorithms were fused using a hierarchical majority vote, leading to overall better performance than any individual algorithm. This finding is particularly relevant to differentiating cystic lesions in the sellar region. AI models may need to be fine-tuned for each lesion type (such as differentiating between cystic and solid tumors with cystic components). By combining multiple AI models, as shown in BRATS, researchers can potentially achieve higher segmentation accuracy than relying on a single model [40]. Additionally, BRATS identified that generative and discriminative models were both applicable but had different strengths; for instance, generative models could incorporate prior knowledge about the appearance of the tumor and were better at generalizing across different datasets, while discriminative models directly learned the relationship between image intensities and segmentation labels. This balance between learning from raw data and incorporating prior domain knowledge could be especially useful for differentiating sellar cystic lesions, which share standard imaging features but require precise and accurate segmentation to aid in diagnosis [40].

Menze et al.'s findings from the BRATS challenge highlight the importance of adopting a multi-model approach in the context of brain lesion differentiation. The significant variability in human annotations of tumor subregions underscores the complexity of such tasks, akin to the challenges encountered in differentiating sellar lesions. The BRATS study demonstrates that no single algorithm consistently performs optimally across all subregions of brain tumors, emphasizing the need for ensemble methods that combine multiple algorithms to achieve better accuracy. This insight directly applies to the differentiation of cystic lesions in the sellar region, where integrating generative and discriminative models could enhance performance and overcome limitations associated with individual AI models [40].

Open Access Series of Imaging Studies (OASIS)

The OASIS dataset study (Kaggle, San Francisco, USA) [41] provides a valuable resource for advancing AI models in neuroimaging, particularly in understanding brain aging and neurodegenerative diseases. The OASIS was created to provide longitudinal MRI data that includes both nondemented and demented older adults. It is a critical tool for studying brain atrophy and its progression in conditions such as Alzheimer’s disease (AD) [41]. The dataset comprises MRI scans from 150 individuals aged 60 to 96, with follow-up imaging sessions occurring at least one year apart. This allows for the tracking of changes in brain structures over time. The data, including metrics like normalized whole-brain volume (nWBV), demonstrated significant atrophy in individuals with AD, who experienced an annual decline in nWBV of 0.87%, compared to 0.49% in nondemented individuals. These findings underscore the utility of longitudinal MRI data in capturing subtle anatomical changes associated with aging and cognitive impairment [41].

This longitudinal approach to understanding brain changes is highly relevant to the goals of this paper, which seeks to differentiate cystic lesions in the sellar region using AI. Although the OASIS dataset focuses primarily on AD and brain aging, its detailed segmentation and processing techniques apply to differentiating other brain pathologies, such as cystic lesions [41]. Tracking brain volume changes over time offers an opportunity to adapt these methods to study lesion progression, potentially distinguishing between benign and malignant cystic lesions in the sellar region. The high contrast-to-noise ratio and multi-session MRI data provided in OASIS offer a strong foundation for developing AI models that require high-quality imaging data for accurate segmentation and classification [41].

A study by Ardekani et al. investigated the presence of sexual dimorphism in the corpus callosum (CC), focusing on the midsagittal CC cross-sectional area (CCA) using MRI data from the OASIS database. Previous studies had presented conflicting conclusions regarding gender differences in the CCA, often due to small sample sizes and inconsistent methodologies. This study aimed to resolve these discrepancies by utilizing a larger sample size and applying advanced statistical controls to account for confounding factors such as brain size and age. The null hypothesis proposed that there would be no difference in CCA between males and females once brain size was controlled.

The study analyzed MRI scans from 316 subjects, including 119 males and 197 females, ranging in age from 18 to 94 years. The researchers employed multiple regression analysis to control for brain size and age. Their findings revealed that females had a significantly larger CCA than males after adjusting for these factors, with a statistical significance of P < 0.03 in the full sample [42]. In a subset of 74 young adults (aged 18-29), matched closely for brain size, the difference between males and females was even more pronounced (P < 0.0005), reinforcing the presence of sexual dimorphism in the CC. The study also found a significant relationship between age and CCA, with the CCA decreasing as age increased and a positive correlation between brain size and CCA [42].

The study's results rejected the null hypothesis, proving that females have a larger CCA than males, even after accounting for brain size and age. These findings not only demonstrate sexual dimorphism but also confirm that CCA decreases with age and is positively correlated with intracranial volume. This comprehensive analysis contributes significantly to understanding gender differences in brain morphology [42].

This study is highly relevant to the paper's goals, as it demonstrates how publicly available MRI datasets, such as OASIS, can effectively investigate structural differences in the brain with high precision and statistical rigor [42]. The automated segmentation methods used for the CC provide a valuable framework for exploring other areas of brain structure, such as cystic lesions in the sellar region [42]. Using multiple regression analysis to control for confounding variables illustrates how statistical approaches can enhance the accuracy of results, which is crucial for developing AI models aimed at differentiating lesions. Furthermore, the emphasis on large sample sizes and rigorous controls aligns with the objectives of building reliable and generalizable AI models for medical imaging applications [42].

The methodology and automated processing pipeline developed in this study offer important insights for improving the precision of segmentation tasks, which is particularly valuable when training AI models for lesion differentiation. By leveraging high-quality MRI data and utilizing public datasets like OASIS, this paper can adopt similar techniques to investigate structural differences in cystic lesions, ultimately improving the AI-based differentiation models and contributing to enhanced diagnostic outcomes in neuroimaging [42].

Discussion

Findings on AI's Role in Diagnosing Sellar Cystic Lesions

The literature reviewed highlights the potential of AI and ML models, specifically CNNs and other DL techniques, in improving the differentiation of cystic lesions in the sellar region of the brain. Lesions such as pituitary adenomas, RCCs, and CPs are challenging to differentiate due to their overlapping imaging characteristics, but AI-driven models have shown promising advancements in diagnostic accuracy [40,41]. Models using transfer learning and ensemble methods have significantly improved, suggesting that these AI tools can outperform traditional neuroimaging techniques [42]. Leveraging advanced segmentation techniques, such as attention mechanisms and multi-scale feature extraction, these models provide better differentiation between lesions by detecting subtle morphological variations [11,12]. Additionally, publicly available datasets like OASIS and BRATS have proven essential for developing and validating AI models, highlighting the importance of large, high-quality datasets in training effective diagnostic models.

AI models based on DL techniques have significantly enhanced diagnostic accuracy by detecting nuances in imaging features that may escape manual interpretation. These findings are consistent with other areas of neuroimaging, such as glioma segmentation in the BRATS dataset [41], where AI models have demonstrated a superior ability to differentiate complex structures. The automatic extraction of features from medical images, without reliance on handcrafted features, allows these models to perform more effectively on complex cases like sellar region lesions [11,12]. Importantly, studies utilizing the OASIS dataset show how controlling for confounding variables like brain size and age creates more accurate and generalizable AI models. As a result, these AI applications hold significant potential for improving diagnostic outcomes by facilitating earlier and more precise diagnoses, which is crucial in preventing complications arising from delayed or incorrect diagnosis [16].

The findings in this paper align with research from other domains of neuroimaging, particularly the segmentation of brain tumors in the BRATS dataset [40]. In glioma studies, AI models successfully distinguished between tumor subregions by enhancing cores and necrotic regions, a challenge similar to differentiating cystic lesions in the sellar region [11,12]. These models’ success highlights the potential for multi-modal imaging techniques, such as MRI combined with other imaging modalities (like CT or functional imaging), to enhance diagnostic precision. Applying ensemble methods in neuroimaging, which involves fusing the outputs of several models, proved to be highly effective for brain tumor differentiation and could be similarly helpful for cystic lesions, where single-model approaches might be insufficient [41]. The OASIS dataset’s longitudinal tracking of brain atrophy further supports the need for similar datasets focusing on cystic lesions to track changes over time and enhance diagnostic accuracy [41].

Clinical Implementation

Integrating AI tools into clinical workflows is essential to ensure these models serve as complementary aids to existing diagnostic processes rather than disrupt them. AI can function as a decision-support tool by providing secondary assessments for radiological evaluations, especially in cases where traditional approaches struggle to distinguish between cystic lesions in the sellar region, such as pituitary adenomas, RCCs, and CPs, due to overlapping imaging characteristics [42]. AI tools can help flag ambiguous cases that require further review, thereby enhancing diagnostic confidence [8]. By pre-analyzing imaging studies and highlighting regions of interest, AI can also improve the efficiency of radiologists. For sellar cystic lesions, this could include providing automated segmentations, estimates of lesion volume, or likelihood scores for different lesion types, which reduces the time radiologists spend on routine tasks and allows them to focus on more complex cases [43].

To effectively integrate AI, it is important to address barriers such as a lack of training in AI technologies, reluctance to trust AI results, or concerns about workflow disruption. The study should explore potential solutions, including incorporating AI education into medical training programs and conducting workshops on interpreting AI-generated outputs [44]. Another key aspect is the explainability of AI models, as many clinicians may be hesitant to trust “black-box” models that do not provide insights into how decisions are made. To foster trust, the study should focus on explainable AI techniques that offer visualizations or explanations for AI decisions, such as heatmaps indicating the regions of an MRI that most influenced a diagnosis [44]. These measures can make AI outputs more interpretable and acceptable to clinicians.

For clinical use, AI models must undergo rigorous validation and regulatory approval processes. The paper should explore how the study’s AI model aligns with current regulatory standards, such as those set by the U.S. Food and Drug Administration (FDA) for software as a medical device (SaMD) [45]. Clinical validation studies demonstrating improved patient outcomes or diagnostic accuracy are necessary for regulatory approval [46]. Additionally, AI tools should integrate seamlessly into existing hospital information systems, such as Picture Archiving and Communication Systems (PACS) and Electronic Health Records (EHRs). The paper could discuss technical requirements for integration, including data interoperability standards like DICOM for medical imaging and HL7 for EHR data exchange, ensuring that AI tools fit into existing systems to minimize workflow disruption [45].

Automation of clinical workflows is another potential benefit of AI integration. For example, if a sellar lesion is detected on an MRI, the AI system could automatically recommend further imaging or lab tests based on the lesion's characteristics, streamlining the diagnostic process [47]. AI could also assist with automated report generation, providing radiologists with a draft report based on AI analysis, thus saving time on documentation. However, it is important to consider the financial aspects, as implementing AI involves not only upfront costs for software and training but also ongoing maintenance expenses. The study should present a cost-benefit analysis weighing these costs against potential savings from reduced diagnostic errors, faster diagnosis, and decreased need for follow-up imaging, which could help justify the adoption of AI tools within hospital budgets [46].

To optimize the integration process, it would be beneficial to involve a multi-disciplinary team that includes radiologists, neurosurgeons, endocrinologists, and ophthalmologists. The paper should outline strategies for engaging these specialists in developing AI protocols, ensuring that the AI models meet the specific needs of each specialty. Once deployed, AI models must be monitored for performance in real-world settings. Establishing a feedback loop where radiologists can report cases where the AI tool made errors or provided inaccurate results will be crucial. This feedback could then be used to retrain the AI model, thus continuously improving its accuracy over time. Continuous performance monitoring ensures that the AI system remains clinically relevant and adaptive to new diagnostic trends.

Limitations 

One limitation of this review is the relative scarcity of literature focusing specifically on AI applications for sellar region cystic lesions [39]. Most research in AI-driven neuroimaging has focused on solid brain tumors or neurodegenerative diseases, with less attention paid to cystic lesions. Additionally, while this review highlights the potential of MRI-based AI models, it may underexplore other imaging modalities, such as CT or functional imaging, which could also play a role in lesion differentiation. Moreover, many studies reviewed rely on small, single-institution datasets, limiting the generalizability of their findings. Further research is needed to address these gaps and explore the broader potential of AI in differentiating sellar region lesions. 

Despite the advancements AI offers, several limitations hinder widespread adoption. First, AI models rely heavily on large, high-quality datasets for training and validation. While datasets like OASIS and BRATS have been instrumental in advancing neuroimaging, there is a need for multi-institutional datasets that specifically focus on cystic lesions in the sellar region. The small sample sizes used in many of the studies reviewed often lead to overfitting, where models perform well on training data but fail to generalize to broader clinical contexts.

While MRI offers excellent soft-tissue contrast, combining it with other imaging techniques could improve the AI model's performance by capturing a broader range of lesion characteristics. For example, integrating MRI with PET could provide both anatomical and functional data, enhancing diagnostic accuracy, especially in ambiguous cases. The study should also address the “black-box” nature of DL models, which can hinder clinician trust due to a lack of interpretability. Emphasizing explainable AI techniques, such as saliency maps or SHapley Additive exPlanations (SHAP), would help clinicians understand how AI reaches specific conclusions, potentially increasing adoption [48]. Moreover, there is a risk of bias in AI model development if the dataset does not adequately represent various demographic groups or lesion types, leading to disparities in diagnostic accuracy. Mitigating this bias involves balancing the dataset across different patient demographics, and lesion subtypes, and applying bias-correction techniques during model training [48].

The AI models' promising performance in controlled settings does not guarantee robustness in real-world clinical environments, where factors such as image quality variations, MRI machine settings, and differences in radiological expertise could impact model performance. Multi-site clinical validation studies are necessary to test the adaptability of the AI models across different settings and ensure consistent diagnostic accuracy [48]. Another issue is the challenge of data annotation and quality, as high-quality, annotated datasets are essential for training accurate AI models [49]. Obtaining consistent labeling, especially for rare lesions, can be difficult, and the study should address how variations in data annotation might affect model performance. Collaborative efforts to standardize annotation guidelines and share high-quality datasets could help mitigate this limitation [49].

The integration of AI into clinical workflows also raises regulatory and ethical concerns, particularly regarding patient data use and the need for model transparency [49]. The study should discuss strategies for ensuring compliance with regulations such as the General Data Protection Regulation (GDPR) and the Health Insurance Portability and Accountability Act (HIPAA). Additionally, the ethical implications of AI-driven decision-making, including its influence on treatment choices, should be considered to support the broader adoption of AI technologies [49].

## Conclusions

This paper underscores the significant potential of AI and ML models, particularly CNNs and DL architectures, in advancing the early and accurate differentiation of cystic lesions in the sellar region, such as pituitary adenomas, RCCs, and craniopharyngiomas. The review of current AI applications in neuroimaging highlights how these models, combined with high-quality datasets like OASIS and BRATS, can overcome traditional diagnostic limitations. AI-driven models have enhanced diagnostic precision by detecting subtle differences in lesion morphology, leading to earlier detection and better-informed clinical decision-making. Despite the advancements, the review also identifies the need for larger, multi-institutional datasets and more interpretable AI models to integrate AI into clinical practice further. The results of this paper suggest that, with continued development and validation, AI can become an indispensable tool in neuroimaging, significantly improving patient outcomes in the diagnosis and management of sellar region cystic lesions. Future research should focus on expanding datasets, improving model interpretability, and exploring multi-modal imaging to realize AI's full potential in clinical neuroimaging.
